# A Computational Study on Temperature Variations in MRgFUS Treatments Using PRF Thermometry Techniques and Optical Probes

**DOI:** 10.3390/jimaging7040063

**Published:** 2021-03-25

**Authors:** Carmelo Militello, Leonardo Rundo, Fabrizio Vicari, Luca Agnello, Giovanni Borasi, Salvatore Vitabile, Giorgio Russo

**Affiliations:** 1 Institute of Molecular Bioimaging and Physiology, Italian National Research Council (IBFM-CNR), Cefalu, 90015 Palermo, Italy; giorgio.russo@ibfm.cnr.it; 2 Department of Radiology, University of Cambridge, Cambridge CB2 0QQ, UK; lr495@cam.ac.uk; 3 Cancer Research UK Cambridge Centre, Cambridge CB2 0RE, UK; 4 LAboratorio di Tecnologie Oncologiche (LATO), Cefalu, 90015 Palermo, Italy; fabrizio.vicari.plus@gmail.com (F.V.); giovanni.borasi@gmail.com (G.B.); 5 Department of Biomedicine, Neuroscience and Advanced Diagnostics (BiND), University of Palermo, 90127 Palermo, Italy; luca.agnello@gmail.com (L.A.); salvatore.vitabile@unipa.it (S.V.)

**Keywords:** MRgFUS, proton resonance frequency shift, temperature variations, referenceless thermometry, RBF neural networks, interferometric optical fibers

## Abstract

Structural and metabolic imaging are fundamental for diagnosis, treatment and follow-up in oncology. Beyond the well-established diagnostic imaging applications, ultrasounds are currently emerging in the clinical practice as a noninvasive technology for therapy. Indeed, the sound waves can be used to increase the temperature inside the target solid tumors, leading to apoptosis or necrosis of neoplastic tissues. The Magnetic resonance-guided focused ultrasound surgery (MRgFUS) technology represents a valid application of this ultrasound property, mainly used in oncology and neurology. In this paper; patient safety during MRgFUS treatments was investigated by a series of experiments in a tissue-mimicking phantom and performing ex vivo skin samples, to promptly identify unwanted temperature rises. The acquired MR images, used to evaluate the temperature in the treated areas, were analyzed to compare classical proton resonance frequency (PRF) shift techniques and referenceless thermometry methods to accurately assess the temperature variations. We exploited radial basis function (RBF) neural networks for referenceless thermometry and compared the results against interferometric optical fiber measurements. The experimental measurements were obtained using a set of interferometric optical fibers aimed at quantifying temperature variations directly in the sonication areas. The temperature increases during the treatment were not accurately detected by MRI-based referenceless thermometry methods, and more sensitive measurement systems, such as optical fibers, would be required. In-depth studies about these aspects are needed to monitor temperature and improve safety during MRgFUS treatments.

## 1. Introduction

Image-guided thermal ablations are increasingly employed in minimally invasive treatments in patients with cancer [1,2,3,4]. In the last decades, a large number of high-intensity focused ultrasound (HIFU) [5,6] devices have been used in oncology to cover a wide range of cancer types, such as prostate [7], bone metastases [8], liver [9], breast [10], thyroid [11], uterine fibroids [12,13], liver and pancreas [14], and brain [15]; as well as psychiatric disorders [16] and essential tremor [17,18].

Considering the imaging modalities that currently guide HIFU treatments, two possible methodologies are available: (i) ultrasound-guided therapeutic focused ultrasound (USgFUS) [19,20], which uses the shift of the echo timing related to the temperature variation of the treated tissues [21]; and (ii) magnetic resonance-guided focused ultrasound surgery (MRgFUS) [22], which leverages the intrinsic dependence of the temperature with respect to some fundamental parameters, such as the apparent diffusion coefficient (ADC) of water molecules, the spin-lattice relaxation time (T1), and the water proton resonance frequency (PRF) [23].

In order to evaluate the incidence and severity of adverse reactions to the USgFUS ablation of uterine fibroids, Chen et al. [24] performed a multicenter, large-scale retrospective study involving 9988 patients with uterine fibroids or adenomyosis. Even though all the required procedures were applied, including skin preparation, 26 of the patients had blisters or tangerine pericarp-like burns in their abdominal skin, and two of them required surgical removal of the necrotic tissue. In [25], a preliminary report on bone metastasis pain-palliation therapy with MRgFUS, an unusual second-degree skin burn occurred on the body side opposite to the transducer position. The authors argued that this accident occurred due to a series of energetically intense sonications that may not have been totally included inside the patient’s body, causing a far-field energy accumulation at the air–skin interface [26,27]. In the case of MRgFUS capsulotomy, safety and clinical efficacy need to be carefully assessed by considering issues related to skull heating [16].

With particular interest in MRgFUS, automated techniques for uterine fibroid MR image segmentation have been recently devised to improve treatment planning [28] and evaluation [29,30], thus increasing the result repeatability and reliability [31]. Importantly, the attention of manufacturers to MRgFUS treatment safety has increased in recent years; therefore, multicenter studies have been performed to propose effective solutions. For instance, a modified clinical MRgFUS fibroid therapy system, called Sonalleve (Philips Healthcare, Vantaa, Finland), was integrated with a 1.5 T magnetic resonance imaging (MRI) scanner (Achieva, Philips Healthcare, Best, The Netherlands). This system directly relied upon a skin-cooling device for the treatment of symptomatic uterine fibroids [32]. In the experiments conducted, involving eight patients, no adverse effects were reported when this cooling device was integrated with the patient table to keep the transducer–patient interface at a fixed temperature of 20 °C.

The aim of this work is to explore the sensitivity of MRI guidance to monitor the temperature increase for patient safety [26,27]. In particular, we simulated the temperature variations in a fibroid treatment on a tissue-mimicking phantom, acquiring temperature measurements using thermal imaging provided by the operating console of the MRgFUS ExAblate 2100 (Insightec Ltd., Carmel, Israel), as well as interferometric optical probes. The temperature maps were obtained using classic PRF and referenceless thermometry methods and compared against the measurements.

## 2. Materials and Methods

**Figure 1:**
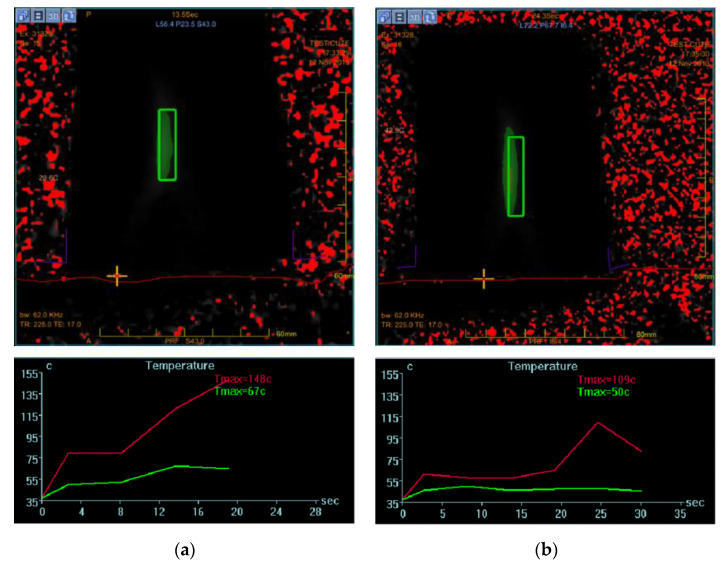
(**a**) Thermal map of a sonication during a treatment. The crosshair cursor, selected by the operator, represents the point of interest for temperature trend control. (**b**) Temperature plot of a single pixel (red line) and a small neighboring region around the crosshair cursor (green line).

In our experiments, an Insightec ExAblate 2100 HIFU transducer integrated with a Signa HTxt MRI scanner (General Electric Medical Systems, Milwaukee, WI, USA) was used. The same clinical device is employed at the Foundation Institute “G. Giglio”, Cefalù (PA), Italy, for uterine fibroid treatment and bone metastasis pain-palliative therapy. This system exploits MRI to acquire temperature maps of treated tissues by quantifying the phase variation resulting from the temperature-dependent changes in the resonance frequency. The phase differences are proportional to temperature-dependent PRF shifts, thus enabling the assessment of temperature rises [33]. Temperature maps derived from MRI can be obtained using gradient recalled echo (GRE) imaging sequences. The console operator monitors the temperature rise taking into consideration: (i) the thermal map of a chosen slice (Figure 1a); and (ii) the temperature plots concerning the selected point (by means of a crosshair cursor) and a small neighboring region (Figure 1b). These methods were successfully used to model the thermal dose delivery [34] strictly related to tissue thermo-ablation [35,36]. Any unwanted temperature increase outside the “target” is due to an energy accumulation, caused by acoustic impedance discontinuity in the ultrasound wave-propagation path [37,38,39].

### 2.1. MRgFUS Treatments

The experimental measurements were carried out using the ExAblate uterine-fibroids protocol, considering a real fibroid treatment as reference.

Prior to MRgFUS treatments for uterine fibroid ablation, the patient was sedated to minimize her movements, but nevertheless she could constantly provide feedback on the perception of pain and heat during the treatment. The MR images were acquired to localize the fibroid position and to plan the treatment with the most suitable ultrasound beam path, and sonication size and number. The treatment was planned by software that analyzed the region of treatment (ROT)—i.e., the region that will undergo the ultrasound beams—and the limited energy density regions (LEDRs)—i.e., the regions containing the organs at risk (OARs). Treatment planning aims to deliver the sonications in the entire ROT, making sure that the ultrasound beam does not cross the LEDRs.

To verify the focus-position accuracy, a preliminary sonication at sublethal energy was delivered. Some MR images were acquired to detect the temperature distribution in the neighborhood of the focus point. Using an iterative procedure, the operator can modify the wave characteristics to improve the target accuracy and the temperature increase. As a result, the treatment was performed by delivering sonications with lethal energy. Each sonication typically lasted 20–40 s, with a cooling time of 80–90 s between two successive sonications.

At the end of the treatment, the patient, in the position she had during treatment, underwent a diagnostic MR examination with gadolinium-based contrast medium, aimed to evaluate the nonperfused volume (NPV), which was the uterine fibroid area covered by sonications. Moreover, the skin was examined to evaluate any side effects due to the temperature increase during the treatment.

**Figure 2:**
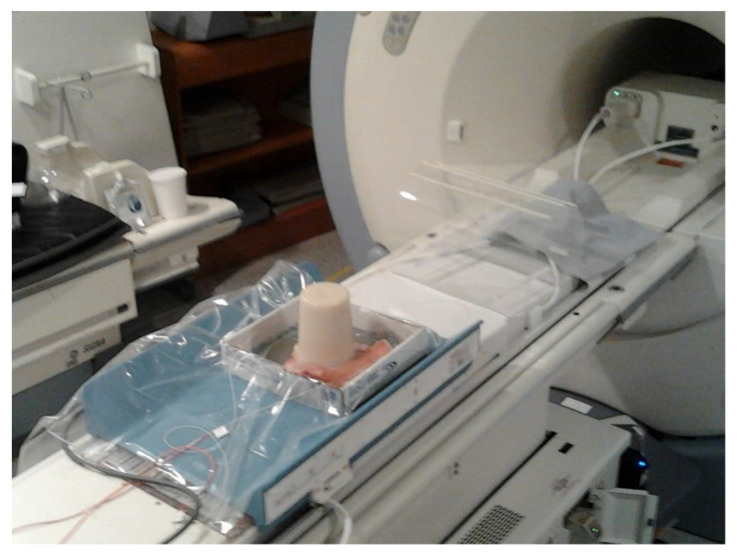
The realized configuration: the daily quality assurance (DQA) phantom over a skin portion. Although barely noticeable, the gel pad was placed under the skin to ensure acoustic coupling between the ExAblate bed and skin. In the left area of the image, the two interferometric probes are visible.

In this work, to quantify temperature increases in the interface area suffering from acoustic impedance discontinuity in the ultrasound wave-propagation path, we used a configuration composed of: (i) a standard phantom tissue mimicking the daily quality assurance (DQA) routine, as previously proposed by Zucconi et al. [40]; (ii) an ex vivo porcine skin sample placed under the phantom to simulate the patient’s skin; and (iii) a gel pad (between the porcine skin and ExAblate bed). A set of interferometric probes was also used to monitor the skin temperature, over the probes and gel pad (Figure 2). We assumed that the porcine skin would respond to the temperature increases like the human skin.

A ROT of 78.7 cm^3^ was defined inside the phantom and automatically covered by the system with 56 sonications. Neglecting absorption and attenuation in the propagation path [41], an average energy of 2353 ± 611 J can be attributed to the sonications emitted by the 208 elements of the phased-array HIFU transducer [42], for an average duration of 20.0 ± 2.9 s (with an elongated beam geometry). The time cooling was set at 85 s and the ultrasound frequency at 1.1 MHz.

The software distributed the sonications over the ROT, forming s-shaped paths, in order to prevent local overheating.

### 2.2. Optical Thermometry

For continuous temperature monitoring during the MRgFUS sonications, an MR-compatible instrumentation was required. The AccuSens interferometric signal conditioner (Opsens Inc., Québec, QC, Canada) equipped with an OTP-M birefringent crystal sensor was chosen. The main characteristics are reported in Table 1.

**Figure 3:**
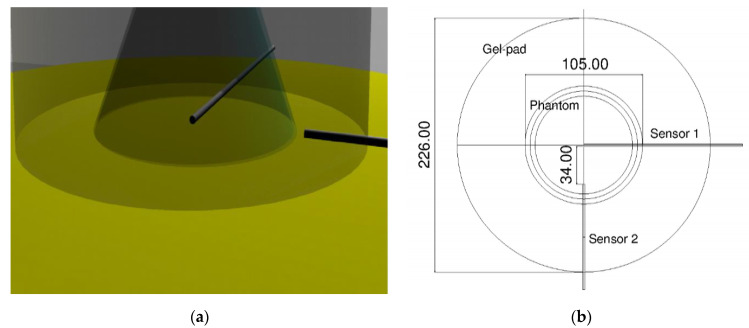
Probe positions relative to the ultrasound field. (**a**) 3D model with the two probes positioned; (**b**) schematics of the positioning/coupling apparatus.

The bottom surface of the phantom was divided into two portions: a circular crown, which was never crossed by the ultrasound, and an inner area covered by the HIFU. One of the OTP-M probes was inserted into the middle of the circular region, and the tip of another one on the boundary between these two regions (Figure 3). Using this configuration, a mask for the relative positioning of sensors and phantom on the gel pad was designed. Then, this mask was reproduced on a plastic drape included in the “patient accessory set” necessary for the treatment, since this material did not introduce any acoustical impedance discontinuity.

### 2.3. Signal-to-Noise-Ratio Estimation

In order to evaluate if there was an adequate signal within the interface region necessary to quantify temperatures, the signal-to-noise ratio (SNR) was calculated according to Gorny et al. [43]. The investigated areas were the phantom, the skin interface, and the gel pad.

Some sample MR images were evaluated; in particular, the images of the phantom relative to sonication 4 and 5 were examined. Each acquired region was characterized by an overall thickness of 16 mm, and was acquired in different locations with respect to the phantom size (circular base with a diameter of 105 mm, as shown in Figure 3).

**Figure 4:**
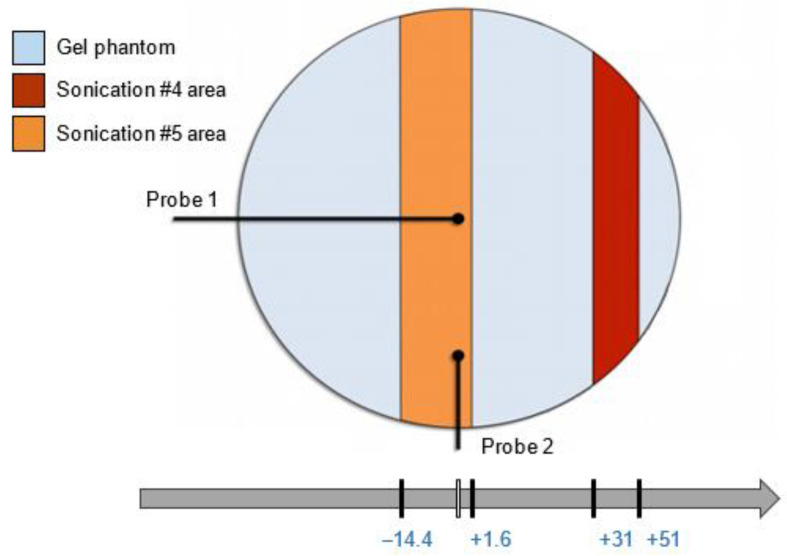
The MRI acquisition locations of sonications #4 and #5.

As shown in Figure 4, the acquired region of sonication #4 (red area) ranged from +35 mm to +51 mm, while the region of sonication #5 (orange area) ranged from −14.4 mm to +1.6 mm.

The images related to each region were acquired in subsequent temporal frames of 3 s, which allowed us to reconstruct the temporal trend of the temperature rise for each acquired area. The SNR value was calculated according to Equation (1):

(1) $$\mathrm{SNR}=\frac{0.655\cdot \mu \left({\mathrm{Signal}}_{\mathrm{object}}\right)}{\sigma \left({\mathrm{Signal}}_{\mathrm{background}}\right)},$$

where the ratio between the mean signal value (μ) of the object (i.e., phantom, skin, and gel pad) region of interest (ROI) and the standard deviation (σ) of an area that contains only background noise (e.g., air) were considered. The 0.655 factor was due to the Rician distribution of the background noise in a magnitude image, which tended to a Rayleigh distribution as the SNR tended to zero [44].

**Figure 5:**
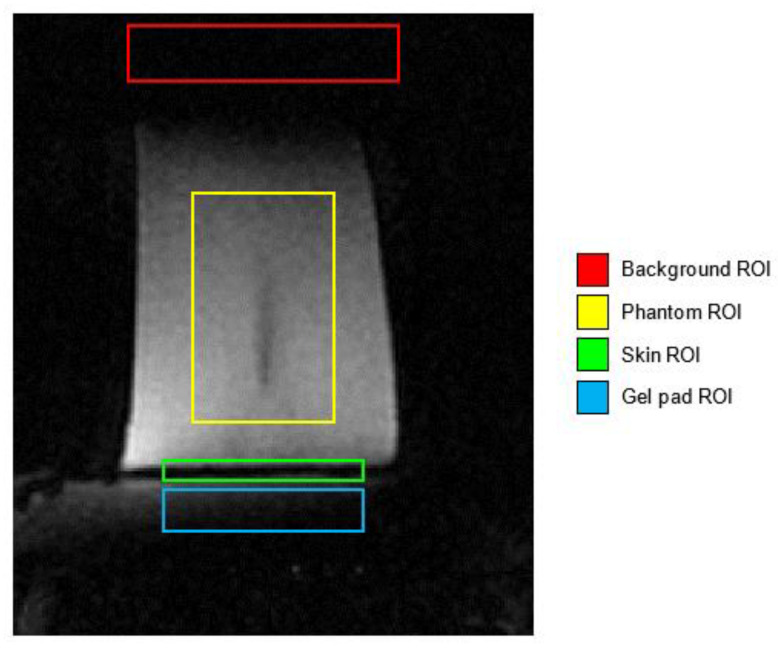
The ROIs investigated for the SNR estimation. The different ROIs that were drawn were the tissue-mimicking phantom (yellow), skin interface (green), gel pad (cyan), and background area (red).

The three ROIs investigated for the SNR estimation are represented in Figure 5. The signal intensity of the phantom, the skin interface, and gel pad areas were compared to a region where the signal was ideally zero (i.e., the background ROI).

### 2.4. Referenceless Thermometry

Classical PRF shift thermometry—in which one or more baseline images are acquired before the thermal therapy and then are subtracted pixel-by-pixel from the images acquired during heating—is affected by artifacts, which could lead to unrealistic temperature increases [13,45,46]. These temperature-independent artifacts are mainly due to movements of the anatomical region undergoing MRgFUS treatments, or to magnetic field inhomogeneities. With the goal of reducing these issues, referenceless thermometry could be used, thus allowing us to estimate the heating caused by an MRgFUS treatment without using a baseline image as temperature reference.

With the goal of accurately estimating the temperature variations, referenceless thermometry methods were developed; in particular, we devised an interpolation method based on artificial neural networks (ANNs) to reconstruct the original baseline phase image and reliably evaluate temperature variations in the sonication area [47,48]. In fact, assuming that the phase image surrounding the treated region has a smooth trend (even under the heated area), referenceless (or self-referenced) thermometry techniques estimate the temperature variations by means of a set of smooth low-order polynomial functions to the surrounding phase, or to a complex magnitude image with the same phase using a weighted least-squares fit [49]. The extrapolation of the polynomial inside the heated region is used as background phase estimation, which is subtracted from the actual phase to evaluate the phase difference before and after heating caused by ultrasound sonications and, successively, quantify the temperature increase.

**Figure 6:**
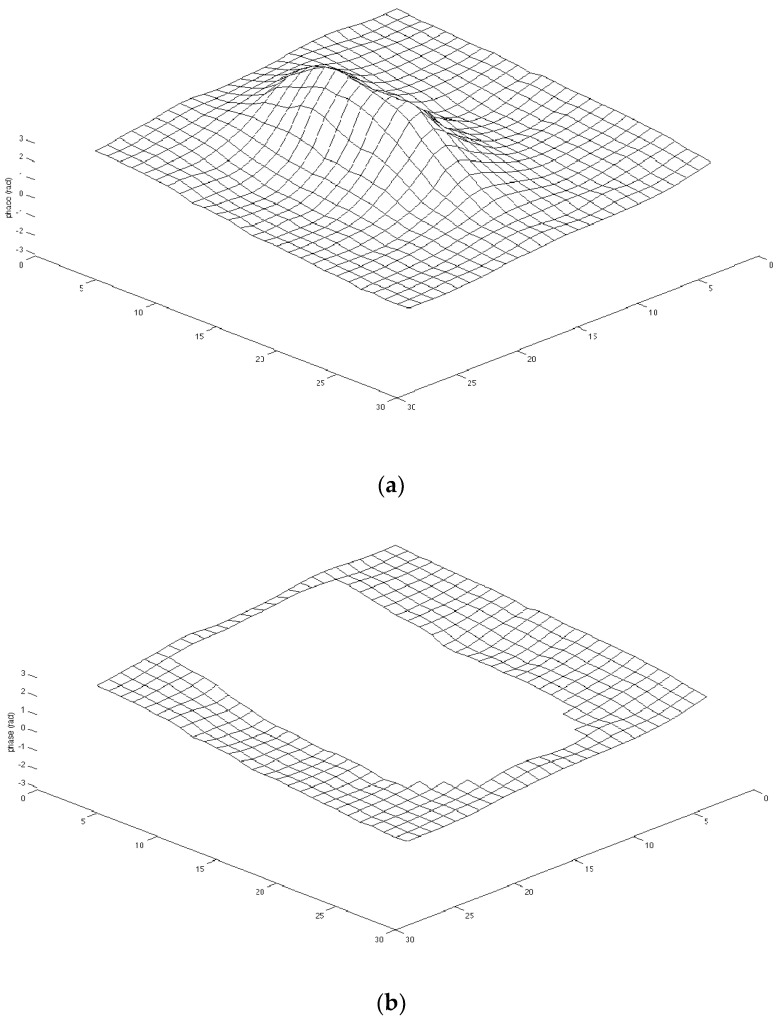
(**a**) 3D plot of a phase map with sonicated area; (**b**) 3D plot of the outer region of the phase map in (**a**) after removing the sonicated area.

In the referenceless phase estimation, an ROI has to be delineated around the area to be heated. First of all, two regions (namely, outer and inner) must be selected in the phase image to perform the interpolation. Figure 6 shows the phase map and the outer baseline region around the sonicated area (after the removal of the inner ROI containing the heated region). It is essential to choose the outer ROI outside the heated region because the temperature changes within the ROI affect the reconstruction of the background phase.

The most straightforward computational approach to solve this problem is to fit the data with a polynomial function [50]. However, an invertible system that uniquely defines the interpolant is not guaranteed for all positions of the interpolation points, and often it could show spurious bumps. The background phase in the frame ROI is reconstructed by means of an ANN exploiting radial basis functions (RBFs) as kernel [51,52].

In particular, a 3-layer feed-forward ANN was designed (with 1 input layer, 1 output layer and 1 hidden layer) in which each hidden node implemented an RBF. ANNs are well-suited for interpolation purposes, especially if there are large areas of missing data, and the RBF approximation method allows several advantages with respect to polynomial interpolants: (i) the network training finds the optimal weights from the input to the hidden layer, and then the weights from the hidden to the output layer are calculated; and (ii) the geometry of the input points is not restricted to a regular grid.

#### Radial Basis Function Theory

Let $f:R^{d}\to R$ be a real valued function of d variables that has to be approximated by $s:\;R^{d}\to R$, given the values $\left\{f\left(X_{i}\right):i=1,2,\ldots ,n\right\}$, where $\left\{X_{i}:i=1,2,\ldots ,n\right\}$ is a set of n distinct points in $R^{d}$ called the interpolation nodes. We will consider an approximation of the form:

(2) $$s\left(X\right)=p_{m}\left(X\right)+\sum_{i=1}^{n}\lambda_{i}\varphi \left(\|X-X_{i}{\|}_{2}\right),\;X\in R^{d},\lambda_{i}\in R,$$

where: $p_{m}$ is a low-degree polynomial that can be also omitted, $\|\cdot {\|}_{2}$ denotes the Euclidean norm, and φ is a fixed function from R to R. Thus, the radial basis function s(·) is a linear combination of translations of the single radially symmetric function $\varphi (\|\cdot {\|}_{2})$, plus a low-degree polynomial. We will denote with $\pi_{m}^{d}$ the space of all polynomials of degree m at most in d variables. The coefficients $\lambda_{i}$, which represent the weights of the approximation s, are determined by requiring that s satisfies the interpolation conditions expressed in the following Equation (3):

(3) $$s\left(X_{j}\right)\equiv f\left(X_{j}\right),\;j=1,2,\ldots ,n,$$

together with the side conditions:

(4) $$\overset{n}{\underset{i=1}{\sum }}\lambda_{i}q\left(X_{j}\right)=0,\;\forall q\in \pi_{m}^{d}.$$

Some typical conditions on the nodes under which the interpolation conditions (3) and (4) uniquely specify the radial basis function (2) are given in Table 2. In this context “not coplanar” means that the nodes do not all lie in a single hyperplane, or equivalently that no linear polynomial in d-variables vanishes at all the nodes. The surveys presented in [53] and [54] are excellent references to these and other properties of radial basis functions.

## 3. Results

**Figure 7:**
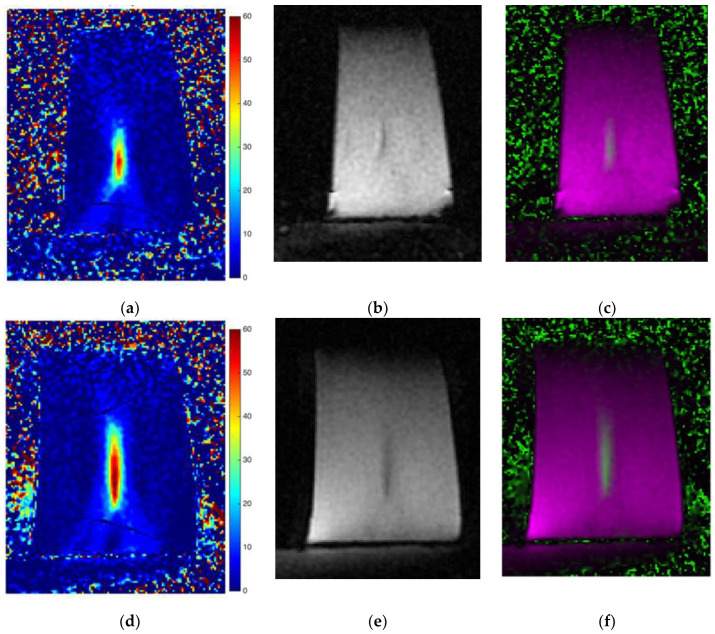
Sonications #4 (first row) and #5 (second row) morphological and thermal map examples: (**a**) and (**d**) temperature reconstruction; (**b**) and (**e**) morphological image; (**c**) and (**f**) temperature image overlapped on the morphological image. It is possible to estimate the noise in the gel pad and in the skin interface by observing the low SNR in those areas.

The selected ROIs were propagated for all the temporal sequences and in all the depths, so the SNR value was calculated on every acquired 3D volume. As depicted in Figure 7, the MR images of the sonications #4 and #5 showed the impulsive noise in the area surrounding the phantom, especially in the skin interface and in the gel pad.

The signal acquired using the thermometric MRI protocol can be acceptable for aqueous tissues (such as the regions treated with MRgFUS), but unsatisfactory for fatty tissues. In fact, as widely stated in [55], the tissue-type temperature independence of the PRF shift is almost true for aqueous tissues, while the dependence in adipose tissues is affected by susceptibility effects. Consequently, the temperature sensitivity of fat is extremely low [56], indicating that MRI-based thermometry inside fatty tissues (such as the skin interface taken into account here) is difficult.

**Figure 8:**
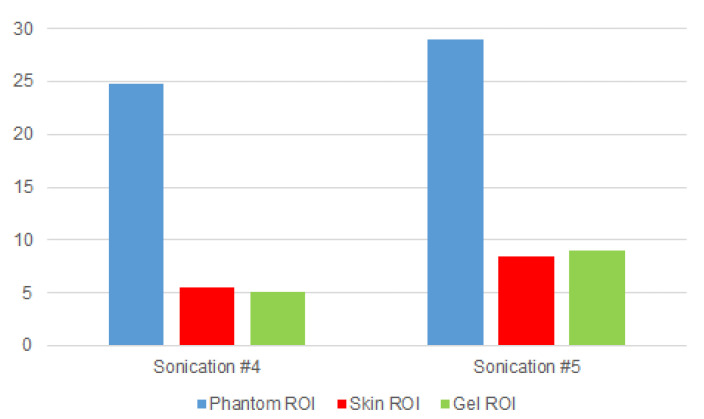
SNR values calculated for the phantom, skin, and gel ROIs. The phantom signal had the highest SNR values, while the gel area and the skin-interface area had the lowest SNR values.

These insights also were confirmed by our experimental findings, which showed that SNRs inside the area near the gel pad and the porcine skin were relatively low when compared to the SNR inside the phantom. Figure 8 shows that the signal was globally low in all three acquired MRI volumes. The phantom area showed a higher signal compared to the skin layer and the gel pad, where the signal appeared very poor.

**Figure 9:**
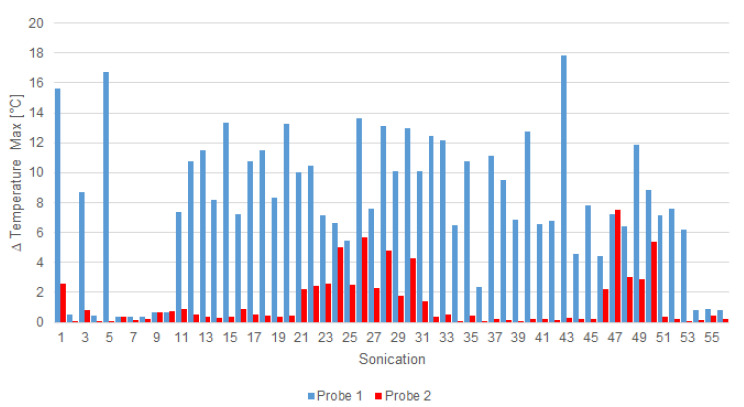
Maximum rise of temperature in each sonication for probe 1 (blue) and probe 2 (red).

The treatment was performed in about 2 h. The interferometric probes under the porcine skin, positioned according to the scheme on Figure 3, measured a large amount of temperature data. Figure 9 shows the maximum temperature rise recorded by the probes in all the sonications. This is a clear confirmation that the probes were actually placed as planned: the first probe was in the middle of the phantom and received more heat than the second one, which was in a more decentralized position than the ROT.

**Figure 10:**
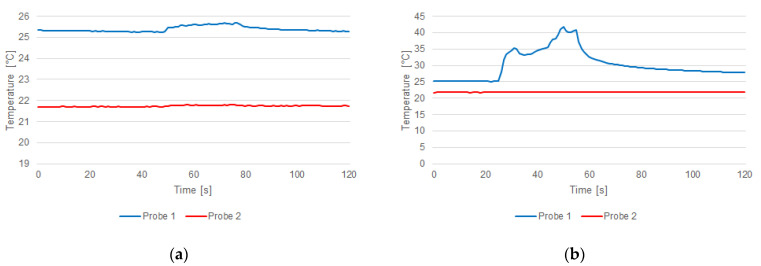
Temperature measured by the optical probe 1 (blue) and probe 2 (red) during sonications #4 (**a**) and #5 (**b**).

In some sonications, temperature-rising measurements were weakly perceived (ΔT < 1 °C) for the relative position along the hypersonic field; this was the case in the fourth sonication (Figure 10a). In other cases, like the fifth sonication, the temperature rose about 16 °C (Figure 10b).

Our analysis, coupled with the PRF-based temperature quantification provided by the ExAblate control console, was employed by considering referenceless thermometry on 2D phase map data, by means of ANNs using different interpolants RBF kernels (i.e., linear, thin-plate spline, and multiquadratic) [47]. In these cases, it also was not possible to detect meaningful temperature increases.

**Figure 11:**
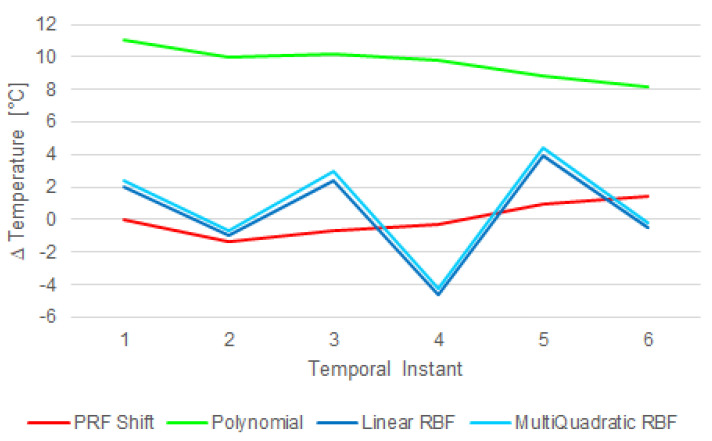
The interpolated temperature errors compared to PRF-based temperature measurements (which does not show any significant temperature rise). The polynomial (green) line overestimated the data, while the linear RBF (blue) and multiquadratic RBF (cyan) lines had a “bump-like” trend caused by the presence of noisy data.

RBF and polynomial interpolations were applied on the data set; the former showed a “bump-like” tendency and the latter overestimated the temperature, because the analyzed area was characterized by a low signal intensity where the noise was a significant component (Figure 11).

To show the differences measured by the two probes, a two-sided Wilcoxon signed rank test on paired data [57] was performed with the null hypothesis that the samples came from continuous distributions with equal medians. In all the tests, a significance level of 0.05 was considered. More details are provided in what follows: (i) the distributions of the temperature increases measured by the probes (Figure 9) were statistically significant considering all the sonications (*p* = 1.719 × 10^−10^); (ii) the distributions of the temperature measured over time by the probes (Figure 10) were statistically significant for sonications for both sonications #4 (*p* = 2.095 × 10^−24^) and #5 (*p* = 6.601 × 10^−44^); and (iii) the polynomial interpolation (Figure 11) significantly overestimated the data (*p* = 0.031), while the linear RBF and multiquadratic RBF interpolations were not statistically different from the PRF shift data (*p* = 0.687 in both cases).

## 4. Discussion

Starting from the current issues concerning patient safety related to undesired temperature variations that can cause skin burns, an MRgFUS fibroid treatment was simulated using an ex vivo porcine skin and a DQA tissue-mimicking phantom. The treatment consisted of 56 ultrasound sonications and a maximum temperature increment (ΔT = 17.78 °C, given in the 43th sonication), as shown in Figure 9. Even if the temperature increase was obtained intentionally through bad acoustic coupling and by considering the interference of the probes, the obtained results showed how it is quite difficult for a clinical operator to detect a possible (and naturally unwanted) temperature increase by relying only on the operating console that displays MR thermographic images. According to the study of Moritz and Henriques [58], the relationship between time and temperature for this sonication is not intense enough to cause a skin burn, but the authors showed how a repetition of five times could lead to complete and irreversible epidermal necrosis. The same results can be obtained using more recent model-based classification approaches [59]. PRF-based temperature monitoring is not useful with this kind of tissue, which was also confirmed by using referenceless thermometry with polynomial and RBF interpolation models. This can be attributed to the small thickness of the skin in the axial and sagittal planes compared to: (i) the spatial resolution of the acquired MR images, (ii) the difficulty of catching the skin on a coronal slice in the low-quality (to guarantee the appropriate acquisition speed for real-time temperature monitoring) MR images acquired during the treatment, and (iii) the thermometry system developed for clinical applications that is not optimized for such a purpose. Moreover, the bump-like tendency of the RBF interpolation errors (see Figure 11) could be due to a low SNR in the analyzed area, where the noise represented a significant component while the signal was practically negligible, as shown in Figure 8.

While attempts have been made to reduce temperature increases on patients’ skin through the quantification of the near-field (between the ultrasound transducer and target) heating [60], a real-time temperature monitoring could give a better control during the treatment. It might be necessary to develop novel image-processing algorithms and methods to enhance phase-map acquisition in PRF-based thermometry techniques, as well as MRI sequences with a higher pixel resolution, to improve the temperature monitoring and limit any unwanted hot spots.

## 5. Conclusions and Future Work

In this work, the potential side effects regarding patient safety due to temperature increases that rarely affect MRgFUS treatments were assessed. Along with the classical PRF shift thermometry, a novel approach that exploited a referenceless technique based on the RBF interpolation was used to evaluate the skin temperature during sonications. Moreover, in this study, we also used two interferometric probes to measure the reached temperatures. In a simulation of a real uterine fibroid treatment, only the probes were able to detect temperature increases, while no important temperature changes were revealed by the used interpolation methods. The achieved results showed that these methods, based on the PRF shift thermometry, could be unsuitable to detect temperature increases on the skin.

One of the issues to consider in our analysis is the low SNR value in the investigated region. New hardware and software solutions need to be studied to increase the temperature-detector sensitivity by rising the SNR in order to also enhance MRgFUS treatment safety and effectiveness.

In the future, more temporal instants should be considered for temperature measurements and increases. Multiple repetitions of the experiments will increase the statistical robustness of the experimental findings.

Moreover, the planned experiments could be designed to reliably simulate a configuration for clinical environments. To address the issues related to the acoustic interference generated by the optical fibers across the ultrasound propagation, other techniques that are able to accurately measure the skin temperature in real time and with a good time resolution could be employed. For instance, thermoscanners have a high temperature accuracy (±0.3 °C), a very high recognition speed (<300 ms), and a temperature range (25–45 °C) that are sufficient to evaluate skin temperature increases in real time. Some systems could be also optically coupled to monitor the skin’s irradiated area for all tests. After extensive ex vivo tests, the developed systems could be employed during clinical treatments.

## Figures and Tables

**Table 1:** Characteristics of the AccuSens interferometric signal conditioner.

Characteristic	Value
Temperature operating range	0 °C to 85 °C
Specific calibrated range	20 °C to 45 °C standard (other ranges available)
Resolution	0.01 °C
Accuracy (specific calibrated range)	±0.15 °C @ ±3.3 σ limit (99.9% confidence level)
Response time	<1 s
Operating humidity range	0–100%

**Table 2:** Conditions imposed on nodes for various radial basis interpolants.

Function Type	Spatial Dimension d	Polynomial Degree m	Restriction on Nodes
linear RBF	any	1	not coplanar
thin-plate spline	2	1	not coplanar
Gaussian	any	absent	none
multiquadratic RBF	any	absent	none

## Data Availability

Data sharing is not applicable to this article.
